# Benzyl and Methyl Fatty Hydroxamic Acids Based on Palm Kernel Oil as Chelating Agent for Liquid-Liquid Iron(III) Extraction

**DOI:** 10.3390/ijms13022148

**Published:** 2012-02-16

**Authors:** Md Jelas Haron, Hossein Jahangirian, Sidik Silong, Nor Azah Yusof, Anuar Kassim, Roshanak Rafiee-Moghaddam, Behnam Mahdavi, Mazyar Peyda, Yadollah Abdollahi, Jamileh Amin

**Affiliations:** 1 Department of Chemistry, Faculty of Science, Universiti Putra Malaysia, 43400 UPM, Serdang, Selangor, Malaysia; E-Mails: sidik@science.upm.edu.my (S.S.); azah@science.upm.edu.my (N.A.Y.); anuar@science.upm.edu.my (A.K.); jaamin2000@yahoo.com (J.A.); 2 School of Chemical Sciences and Food Technology, Faculty of science and Technology, Universiti Kebangsaan Malaysia, 43600 UKM Bangi, Selangor, Malaysia; E-Mails: roshanak.rafiee@gmail.com (R.R.-M.); behnammahdavi@yahoo.com (B.M.); 3 Department of Chemical and Environmental Engineering, Faculty of Engineering, Universiti Putra Malaysia, 43400 UPM, Serdang, Selangor, Malaysia; E-Mail: mazyarpeyda@yahoo.com; 4 Advanced Materials and Nanotechnology Laboratory, Institute of Advanced Technology, Universiti Putra Malaysia, 43400 UPM, Serdang, Selangor, Malaysia; E-Mail: yadollahabdolla@putra.upm.edu.my

**Keywords:** Iron extraction, methyl fatty hydroxamic acid, benzyl fatty hydroxamic acid, iron(III) methyl fatty hydroxamate, iron(III) benzyl fatty hydroxamate, palm kernel oil

## Abstract

Liquid-liquid iron(III) extraction was investigated using benzyl fatty hydroxamic acids (BFHAs) and methyl fatty hydroxamic acids (MFHAs) as chelating agents through the formation of iron(III) methyl fatty hydroxamate (Fe-MFHs) or iron(III) benzyl fatty hydroxamate (Fe-BFHs) in the organic phase. The results obtained under optimized conditions, showed that the chelating agents in hexane extract iron(III) at pH 1.9 were realized effectively with a high percentage of extraction (97.2% and 98.1% for MFHAs and BFHAs, respectively). The presence of a large amount of Mg(II), Ni(II), Al(III), Mn(II) and Co(II) ions did affect the iron(III) extraction. Finally stripping studies for recovering iron(III) from organic phase (Fe-MFHs or Fe-BFHs dissolved in hexane) were carried out at various concentrations of HCl, HNO_3_ and H_2_SO_4_. The results showed that the desired acid for recovery of iron(III) was 5 M HCl and quantitative recovery of iron(III) was achieved from Fe(III)-MFHs and Fe(III)-BFHs solutions in hexane containing 5 mg/L of Fe(III).

## 1. Introduction

Separation and concentration of metal ions including iron(III) are important from the perspective of both environmental and economic profits. Many reagents have been used for extraction and separation of iron(III) from aqueous solutions [1]. Recently Rios *et al.* reported an unconventional solvent less method for iron(III) extraction using ionic liquids. They reported that the ionic liquid methyltrioctylammonium chloride allowed almost complete extraction of iron(III) from the aqueous solutions [2]. Although the results of the iron(III) extraction with ionic liquids as an alternative to the traditional liquid-liquid extraction is very promising, they suffer from some drawbacks such as toxicity and high cost of the reagent [3]. Hence many workers still use the conventional methods which use chelating reagents to chelate metal ions and extraction into organic solvents. Jayachandran *et al.* studied the liquid-liquid extraction of iron(III) with 2-ethyl hexyl phosphonic acid mono 2-ethyl hexyl ester (PC-88A) in toluene. They showed that quantitative extraction of iron(III) with 5 × 10^−3^ M PC-88A in toluene was achieved in the pH range 0.75–2.5. From the extracted complex species in the organic phase, iron(III) was stripped with 1–4 M HNO_3_, 1.5–4 M H_2_SO_4_ and 1.5–4 M HCl. Finally they extended the method for the determination of iron in real samples [4]. Saji and Reddy used a mixed solvent system consisting of tributyl phosphate and methyl isobutyl ketone for extraction of iron(III) from hydrochloric acid solution. The results demonstrate that iron(III) is extracted as HFeCl_4_ with 2 mol of the solvent. The potential of the mixed solvent system for the recovery of high purity iron(III) chloride from waste chloride liquors of titanium minerals in the processing industry has been assessed [5].

Hydroxamic acids and their derivatives have been known as metal chelating compounds for more than a half century. The compounds were applied for organometal synthesis, spectroscopic analysis and metal extraction [6–15]. Chiarizia *et al.* studied the extraction kinetics of iron(III) at biphasic medium [16]. They investigated the forward and reverse extraction rate of Fe^3+^ at time zero between aqueous nitrate solutions and toluene solutions of tri-*n*-butylacetohydroxamic acid, and showed that the extraction reactions occur simultaneously in the aqueous phase (homogeneous path) and at the interface (heterogeneous path) and also correlation between the rate constants and the equilibrium constant of the extraction reaction of Fe(III) has been established. Afeworki and Chandravanshi innovated simple, precise, sensitive, and highly selective methods for the separate determination of iron(III) and cobalt(II) and for the simultaneous determination of both metal ions [17]. They showed that iron(III) and cobalt(II) react with thiocyanate in the presence of *N*-phenylcinnamohydroxamic acid to form pinkish red and blue colored complexes, respectively and are quantitatively extractable into ethylacetate from 0.5–1.5 M hydrochloric acid solutions. They studied the effects of foreign ions and various experimental parameters to optimize the conditions for the extraction and used the methods in the analysis of blood, vitamin B_12_, and standard steels for iron and cobalt successfully. Birus and Van Eldik studied the effect of pressure on the complex formation and aquation kinetics of iron(III) with many hydroxamic acids[18]. Agrawal *et al.* reported a new functionalized calyx[6]crown hydroxamic acid for the speciation, liquid-liquid extraction, sequential separation and trace determination of Cr(III), Mo(VI) and W(VI) [19]. They investigated effect of different parameters such as pH/M HCl, time, temperature and concentration of calyx[6]crown hydroxamic acid on the extraction and obtained optimum condition for this purpose. They also obtained suitable extracting organic solvent and determined the stoichiometry of the complex. Chromium(III), molybdenum(VI) and tungsten(VI) were extracted at pH 4.5, 1.5 M HCl and 6.0 M HCl, respectively with calixcrown hydroxamic acid in chloroform in the presence of a large number of cations and anions.

In this study we applied MFHAs and BFHAs based on palm kernel oil as chelating agent for extraction of Fe^3+^ from aqueous media by using liquid-liquid extraction. This is the first report of using fatty hydroxamic acids derivatives as iron chelating agents. The synthesis of MFHAs and BFHAs from palm kernel oil is simple, environmentally friendly and uses easily available palm kernel oil as one of the raw materials [20].

## 2. Results and Discussion

### 2.1. Organic Solvent Selection and Volume Ratio Org/Aq Phases Determination

Different kinds of organic solvent such as heptane, hexane, petrolum ether, xylene and chloroform were applied on iron(III) extraction by MFHAs as chelating agent. Since our aim was to evaluate the ability of MFHAs to extract iron(III), we choose organic solvents that do not have extraction properties for the metal ion. Table 1 shows that heptane gave the highest percentage of iron(III) extraction followed by hexane and xylene gave the lowest. The high percentage of iron(III) extraction in heptane and hexane could be due to MFHAs having linear alky branch which is more soluble in hydrocarbon compared to other types of organic solvents. However due to the cheaper cost of hexane compared to heptane, and the percentage of iron(III) extraction differing only by 0.3 %, hexane was selected as solvent in the subsequent experiment. In order to select the optimum volume ratio of organic aqueous phase, various volumes of hexane containing 3500 mg/L MFHAs were used to extract iron(III) from 50 mL aqueous solution containing 100 mg/L iron(III). It was found that (data not shown) the highest percentage of extraction was achieved when the volume ratio of org/aq is equal to 1 (50 mL org/50 mL aq).

### 2.2. Effect of pH on Iron(III) Extraction

Rydberg *et al.* [21] described the equation of liquid-liquid metal extraction by chelating agents can be written by Equation 1:

(1) $${\text{Fe}}_{\left(\text{aq}\right)}^{3+}+{\text{xHL}}_{\left(\text{org}\right)}\overset{{\text{K}}_{\text{ex}}}{⇄}{\text{FeL}}_{\text{x}(\text{org})}+{\text{xH}}_{\left(\text{aq}\right)}^{+}$$

**Figure 1:**
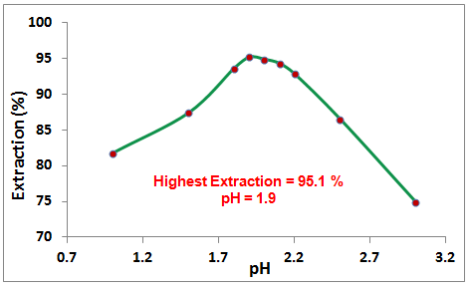
Effect of pH on the iron(III) extraction by MFHAs: [Fe^3+^]_(aq)_ = 100 mg/L, [MFHAs]_(org)_ = 3500 mg/L, pH_(aq)_ = variable.

**Scheme 1:**
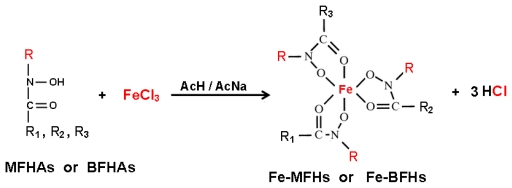
The equation for reaction of iron(III) complexation with fatty hydroxamic acids derivatives: R = methyl, or benzyl. R_1_, R_2_, R_3_ = alkyl branches of different acyl groups obtained from palm kernel oil [20].

**Scheme 2:**
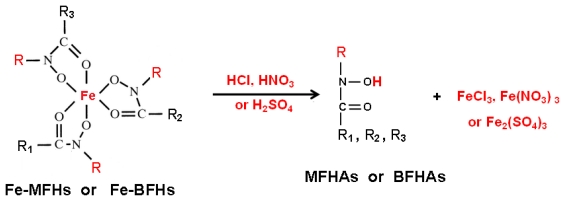
The reaction equations of iron(III) stripping from iron(III) fatty hydroxamate derivatives: R = methyl or benzyl. R_1_, R_2_, R_3_ = alkyl branches of different acyl groups obtained from palm kernel oil.

Where HL, FeL_x_, x and K_ex_ are chelating agent, iron complex, mole ratio of chelate to Fe^3+^ in the complex, and the equilibrium constant of the reaction, respectively. This equation shows iron extraction depend on aqueous pH ([H^+^]_aq_) so effect of pH on the iron(III) extraction was carried out in different pH (1.0, 1.5, 1.8, 1.9, 2.0, 2.1, 2.2, 2.5 and 3.0) of aqueous phase while other parameters were kept constant. The result in Figure 1 shows that pH 1.9 gives the highest percentage of extraction. The down curve at pH above 1.9 could be due to the decrease of solubility of Fe^3+^ while the down curve at pH lower than 1.9 could be due to proton exchange reaction as shown in schemes 1 and 2 (decreasing concentration of MFHs (methyl fatty hydroxamates) anions). This fact is supported by Equation 1 which shows that increasing [H^+^] (decreasing the pH) caused the equilibrium reaction move to the opposite direction of complex formation.

### 2.3. Effect of Chelate Concentration

**Figure 2:**
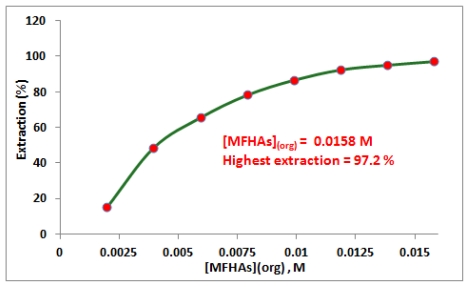
Effect of MFHAs concentration on the iron(III) extraction: [Fe^3+^]_(aq)_ = 100 mg/L, pH_(aq)_ = 1.9.

For this purpose, different concentrations of MFHAs solutions in hexane were used for iron(III) extraction. The results show that the percentage of iron(III) extraction increases when MFHAs concentration in organic phase increases (Figure 2) and maximum iron(III) extraction was obtained when the concentration of MFHAs concentration was 0.0158 M. Higher concentrations of MFHAs were not investigated due to solubility limit of the chelating agent in the solvent.

### 2.4. Determination of Mole Ratio MFHs/Fe^3+^ in Fe-MFHs

**Figure 3:**
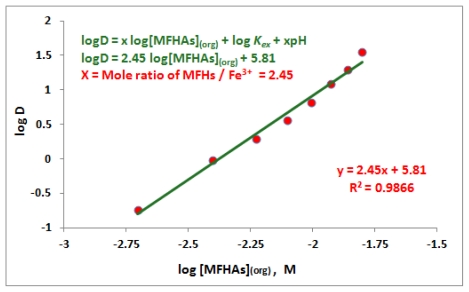
Curve of log D *versus* logarithm of MFHAs concentration in hexane: [Fe^3+^]_(aq)_ = 100 mg/L, pH_(aq_) = 1.9.

From the equilibrium Equation 1, the equilibrium constant, K_ex_ can be written as in Equation 2 below. By defining a distribution ratio, D, as in Equation 3 and transferring it into Equation 2, K_ex_ can be written as in Equation 4 where its logarithm form is given in Equation 5. According to Equation 5 the mole ratio, x, of the MFHAs to Fe^3+^ in the complex can be determined from the slope of the plot log D *versus* log [MFHAs]. The data of the effect of MFHAs concentrations on iron(III) extraction in the previous section were used to plot log D *versus* log [MFHAs] shown in Figure 3. The figure shows that the mole ratio of MFHAs to Fe^3+^ in the complex is equal to 2.5. This result indicates that mixture of complexes with mole ratios 2 and 3 of MFHAs per mole of iron(III) were formed at pH = 1.9. This result is in agreement with that of Farkas *et al*. [22] that described the mole ratio of monohydroxamic acids ligand per mole of iron(III) are dependent on pH and also indicated that the mixture of different species of complexes with different mole ratios of ligand per metal could exist in a specific pH value.

(2) $${\text{K}}_{\text{ex}}=\frac{{\left[{\text{FeL}}_{\text{x}}\right]}_{\left(\text{org}\right)}{\left[{\text{H}}^{+}\right]}_{\left(\text{aq}\right)}^{\text{x}}}{{\left[{\text{Fe}}^{3+}\right]}_{\left(\text{aq}\right)}{\left[\text{HL}\right]}_{\left(\text{org}\right)}^{\text{x}}}$$

(3) $$\text{D}=\frac{{\left[{\text{FeL}}_{\text{x}}\right]}_{\left(\text{org}\right)}}{{\left[{\text{Fe}}^{3+}\right]}_{\left(\text{aq}\right)}}$$

(4) $${\text{K}}_{\text{ex}}=\frac{\text{D}{\left[{\text{H}}^{+}\right]}_{\left(\text{aq}\right)}^{\text{x}}}{{\left[\text{HL}\right]}_{\left(\text{org}\right)}^{\text{x}}}$$

(5) $$\text{log D}=\text{x log }{\left[\text{HL}\right]}_{\left(\text{org}\right)}+\text{log }{\text{K}}_{\text{ex }+}+\text{x pH}$$

### 2.5. Comparison of Iron Extraction by MFHAs and BFHAs

Previous results show that the optimum conditions for extraction of iron(III) from aqueous solution containing 100 mg/L Fe^3+^ by MFHAs are as follows: [MFHAs]_(org)_ = 0.0158 M, pH of aqueous solution = 1.9 and V_org_/V_aq_ = 50 mL/50 mL. These conditions were applied for comparing iron(III) extraction by MFHAs and BFHAs. The results showed that the percentage extraction for MFHAs and BFHAs were found to be 97.2% and 98.1%. A small difference on iron(III) extraction ability between MFHAs and BFHAs ligands could be due to a slight difference in ligands solubility or dissociation to anions due to small differences in electron affinity or steric effect of methyl and benzyl groups.

### 2.6. Iron(III) Separation by MFHAs and BFHAs

In the liquid-liquid extraction, the ability of an extractant to separate a metal ion from another metal ion is measured by a separation factor, SF which is given by Equation 6:

(6) $${\text{SF}}_{\left(\text{M}/\text{N}\right)}={\text{D}}_{\text{M}}/{\text{D}}_{\text{N}}$$

Where D_M_ is the distribution ratio of metal M and D_N_ is the distribution ration of metal N [23]. The results of iron(III) extraction from aqueous solution containing 100 mg/L Fe^3+^ in the presence of similar concentrations of either Mg(II), Ni (II), Al (III), Mn (II) or Co (II) show that the values of SF were higher than 10,000 for both MFHAs and BFHAs ligands. This indicates that the fatty acid derivatives could be effectively used to separate iron(III) from those metal ions. Iron(III) extraction by MFHAs and BFHAs from aqueous solution containing 100 mg/L of Fe(III) in the absence and in the presence of 800 mg/L of metal ions such as Mg(II), Ni(II), Al(III), Mn(II) and Co(II) were carried out. The results in Table 2 show that the presence of the above mentioned ions at high concentration did not significantly affect the percentages of iron(III) extraction.

### 2.7. Iron(III) Stripping and Preconcentration

Equation 1 in Section 2.2 shows that in solvent extraction the formation of iron(III) complexes in the organic phase is dependent on the aqueous pH or H^+^ concentration, indicating that the stability of the complexes in the organic phase can be reduced by decreasing the aqueous pH. Accordingly, for iron(III) stripping from Fe-MFHs and Fe-BFHs in the organic phase to the aqueous phase can be achieved by using a strong mineral acid solution. Iron(III) stripping was then carried out by mixing 50 mL organic phase (hexane) containing iron(III) fatty hydroxamate derivatives with the same volume of different acids at various concentrations separately (V_or_/V_aq_ = 1). The initial iron(III) concentration in organic phase is equal to 95 mg/L. The mixture was stirred at 500 rpm for 10 minutes at 25 ± 1 °C. The results in Table 3 show that the highest recovery of iron(III) was obtained by 5 M hydrochloric acid. This result can be due to easier formation of FeCl_3_ compared to Fe(NO_3_)_3_ and Fe_2_(SO_4_)_3_ which can be due to differences in the steric effect of anions, Cl^−^, NO_3_^−^ and SO_4_^2−^ for formation of the mentioned salts in aqueous phase. Also, the recovery percentage from Fe-MFHs (99.0%) is higher than that of Fe-BFHs (97.9%). This may be due to a lower stability of Fe-MFHs compared to Fe-BFHs in HCl medium. In the next experiment, the effect of volume ratio of organic phase per aqueous phase (5 M HCl) in the stripping process was investigated. Different volume ratio of org/aq such as 4/1, 3/1, 2/1, 1/1 and 1/2 were applied for iron(III) stripping. The results in Table 4 show that highest recovery percentages were obtained while volume ratio of org/aq was 1/2. This result is expected because with increasing the volume of aqueous phase, the amount of proton increases too and consequently stripping will be more effectively completed. Finally, the effect of initial concentrations of iron(III) in the organic phase, on the stripping was investigated using the different concentrations of Fe(III) (in organic phase) such as 95, 50, 25, 10, 5 mg/L while other conditions were kept constant. The results in Table 5 show that the quantitative recovery was achieved as [Fe(III)]_(org)_ was 5 mg/L for both Fe-MFHs and Fe-BFHs.

## 3. Experimental

### 3.1. Material and Apparatus

Sodium acetate, iron(III) chloride, *N*-methylhydroxylamine hydrochloride, and *N*-benzyl-hydroxylamine hydrochloride were purchased from Aldrich (USA). Hexane, heptane, chloroform, petroleum ether, xylene, hydrochloric acid, sulphuric acid, nitric acid and sodium hydroxide were supplied by Systerm Co. (Malaysia). Lipozyme TL IM was purchased from Novo Nordisk (Denmark). Commercial palm kernel oil was supplied by Malaysian Palm Oil Board (MPOB, Malaysia). The standard solutions of metal ions for AAS measurements were prepared by diluting the Spectrosol stock solution from BDH Chemical (England). Atomic absorption spectrophotometer (AA-680 Shimadzu, Japan) with an air-acetylene flame was used for the metal ion analyses.

### 3.2. Synthesize of Chelating Agent

MFHAs and BFHAs were separately synthesized from palm kernel oil and *N*-methylhydroxylamine hydrochloride, or *N*-benzyl-hydroxylamine hydrochloride catalyzed by Lipozyme TL IM according to the method reported recently by our group [20,24]. The molecular weight of MFHAs and BFHAs were 264 and 358 g/mol, respectively as determined from elemental analysis data as reported earlier [20].

### 3.3. General Iron(III) Extraction Procedure

A known amount of each fatty hydroxamic acids derivatives dissolved in 50 mL of an organic solvent was mixed with 50 mL aqueous solution of known concentration of iron(III) nitrate solution buffered by sodium acetate at desired pH (pH = 1.8 to 2.1). The mixtures were stirred at 500 rpm at 25 ± 1 °C for 10 minutes. Finally the organic phase was separated from aqueous phase and the amounts of iron(III) in the two phases were determined by atomic absorption spectrophotometer (AAS).

For optimization of iron(III) extraction, firstly the MFHAs was selected as a chelating agent and the effects of kind of organic phase, ligand concentration in organic phase, aqueous phase pH and volume ratio of organic/aqueous phases were evaluated. The optimized conditions for iron(III) extraction by MFHAs were applied to iron(III) extraction by BFHAs. The extraction percentages were calculated from the following formula (Equation 7):

(7) Extraction (%)=A×100/B

Where A = final amount of iron(III) in the organic phase and B = initial amount of iron(III) in the aqueous phase. Scheme 1 shows the equation of chemical reaction of iron complexation of fatty hydroxamic acids derivatives.

### 3.4. Iron(III) Stripping Study

The chemical reaction of iron(III) stripping is the opposite of iron(III) extraction reaction where an analyte usually is extracted back into a mineral acid. For optimizing the iron(III) stripping, various parameters such as effect of kind and concentration of mineral acid, effect of volume ratio of organic phase per aqueous phase and effect of initial iron(III) concentration in organic phase were investigated. The recovery percentage of iron(III) stripping was calculated by the Equation 8:

(8) Recovery (%)=A×100/B

Where A = Final amount of iron(III) in aqueous phase, and B = Initial amount of iron(III) in organic phase. Scheme 2 shows the proposed equation of chemical reaction of iron(III) stripping from iron(III) fatty hydroxamate derivatives into acid solution.

## 4. Conclusions

The MFHAs and PFHAs based on palm kernel oil are excellent chelating agents for extraction of iron(III) from aqueous mixtures. Among the advantages of using this product as ligand are its simple preparation and the use of vegetable oil (palm kernel oil) as raw material is cheap and readily available. The highest percentage of iron(III) extraction by MFHAs and BFHAs were 97.2% and 98.1%, respectively. Separation factor (RF) for Fe(III) extraction from Co(II), Ni(II), Mn(II), Mg(II), and Al(III) for both MFHAs and BFHAs were higher than 10,000 which indicates that the iron(III) can be effectively separated from those ions. The presence of the above mentioned metal ions at a high concentration did not significantly affect the percentages of iron(III) extraction. The optimum conditions for extraction of 100 mg/L iron(III) with 0.0158 M fatty hydroxamic derivatives were V_org_/V_aq_ = 50 mL/50 mL and at pH_(aq)_ 1.9. Iron(III) ion from Fe-MFHs or Fe-BFHs in 25 mL hexane containing 5 mg/L Fe(III) can be quantitatively stripped into 50 mL HCl (5M) aqueous solution.

## Figures and Tables

**Table 1:** Effect of various organic solvents on the iron(III) extraction by methyl fatty hydroxamic acids (MFHAs): [Fe^3+^]_(aq)_ = 100 mg/L, [MFHAs]_(org)_ = 3500 mg/L, pH_(aq)_ = 3.0, V_org_ = 50 mL, V_aq_ = 50 mL.

Organic Solvent	Final [Fe^3+^](aq) (mg/L)	Extraction (%)
**Heptane**	**24.8**	**75.2**
**Hexane**	**25.0**	**75.0**
**Petrolum ether**	**38.57**	**61.5**
**Chloroform**	**35.5**	**64.5**
**Xylene**	**49.9**	**50.1**

**Table 2:** Effect of foreign ions on the iron(III) extraction by MFHAs and BFHAs ligands: [Fe^3+^]_(aq)_ = 100 mg/L, [foreign metal ions]_(aq)_ = 800 mg/L, [MFHAs]_(org)_ = [BFHAs]_(org)_ = 0.0158 M, pH_(aq)_ = 1.9. Ex. = Extraction, SD = standard deviation.

Metal Ions	MFHAs Ex. (%) ± SD	BFHAs Ex. (%) ± SD
**None**	**95.1 ± 0.4**	**96.2 ± 0.6**
**Mg(II)**	**94.8 ± 0.4**	**95.9 ± 0.6**
**Ni(II)**	**94.9 ± 0.5**	**95.9 ± 0.8**
**Al(III)**	**94.9 ± 0.5**	**96.1 ± 0.4**
**Mn(II)**	**94.6 ± 0.6**	**95.8 ± 0.7**
**Co(II)**	**94.6 ± 0.5**	**95.7 ± 0.5**

**Table 3:** Effect of type and concentration of mineral acids on the iron(III) stripping from Fe-MFHs and Fe-BFHs: initial [Fe^3+^]_(org)_ = 95 mg/L, V_org_/V_aq_ = 50 mL/50 mL. Rec. = recovery.

Acids	Conc. (M)	Fe (% Rec.) Fe-MFHs	Fe (% Rec.) Fe-BFHs
**HCl**	**1 (M)**	**60.5**	**-**
	**3 (M)**	**94.1**	**-**
	**5 (M)**	**99.0**	**97.9**

**HNO_3_**	**1 (M)**	**27.5**	**-**
	**3 (M)**	**55.7**	**-**
	**5 (M)**	**70.3**	**73.7**

**H_2_SO_4_**	**1 (M)**	**29.0**	**-**
	**3 (M)**	**56.9**	**-**
	**5 (M)**	**79.7**	**86.1**

**Table 4:** Effect of volume ratio of organic phase per aqueous phase on the iron(III) stripping of Fe-MFHAs and Fe-BFHAs: initial [Fe^3+^_(org)_] = 95 mg/L, aqueous phase = HCl (5M), Rec. = recovery.

Volume Ratio (org/aq)	Fe (% Rec.) Fe-MFHs	Fe (% Rec.) Fe-BFHs
**4/1**	**71.3**	**70.1**
**3/1**	**89.7**	**86.6**
**2/1**	**97.2**	**93.5**
**1/1**	**99.1**	**97.9**
**1/2**	**99.1**	**98.3**

**Table 5:** Effect of initial iron(III) concentration in the organic phase on the iron(III) stripping of Fe-MFHAs and Fe-BFHAs: V_org_/V_aq_ = 25 cc/50 cc = 1/2, aqueous phase = HCl (5M), Rec. = recovery, SD = standard deviation.

Initial [Fe^3+^]_(org)_ mg/L	Fe (% Rec. ± SD) Fe-MFHs	Fe (% Rec. ± SD) Fe-BFHs
**95**	**99.1 ± 0.4**	**98.3 ± 0.6**
**50**	**99.8 ± 0.3**	**98.8 ± 0.5**
**25**	**99.9 ± 0.6**	**99.1 ± 0.3**
**10**	**99.9 ± 0.5**	**99.6 ± 0.4**
**5**	**100 ± 0.2**	**100 ± 0.2**
